# New host factors and pathways involved in CD4 downregulation in HIV-1 infected cells

**DOI:** 10.1186/1742-4690-10-S1-P45

**Published:** 2013-09-19

**Authors:** Alessia Landi, Jolien Vermeire, Veronica Iannucci, Hanne Vanderstraeten, Evelien Naessens, Mostafa Bentahir, Bruno Verhasselt

**Affiliations:** 1 Department of Clinical Chemistry, Microbiology, and Immunology. Ghent University, Ghent, Belgium; 2 Centre de Technologies Moléculaires Appliquées, Ecole de Santé Publique, Brussels, Belgium

## Background

Downregulation of the CD4 receptor is one of the hallmarks of HIV infection. The virus has evolved redundant mechanisms to remove the receptor from the cell surface and accelerate its degradation, mainly mediated by three viral proteins: Vpu, Env and Nef. We were interested in the discovery of pathways and human proteins involved in the process, which eventually could represent new drug targets.

## Materials and methods

A genome-wide short-hairpin RNA (shRNA) screening using a SBI shRNA lentiviral interference delivery system library compatible with the GeneChip^®^ Human Genome U133 Plus 2.0 Array (Affymetrix) was performed in HeLa CD4^+^ cells expressing the Nef protein introduced by retroviral transduction. CD4 surface levels were measured by flow cytometry.

The read-out in the screen showed the rescue of the CD4-high phenotype despite Nef expression. shRNA sequences enriched in the CD4-high cells compared to the CD4-low cells were identified and filtered via pathway analysis. For the confirmation and further selection of the hits two cell lines in different conditions were used:

1. HeLa CD4^+^ cells expressing Nef after retroviral transduction (similar to previous screening effort).

2. SupT1 lymphocytic cells infected with replication competent HIV-1 encoding a GFP reporter.

3. SupT1 cells expressing Nef or Vpu after retroviral transduction.

In all three experimental set-ups, the cells were selectively knocked-down for each of the hits individually after transduction with a different set of shRNA encoding lentiviral vectors (Mission Consortium, Sigma Aldrich) prior to HIV-1 infection or retroviral transduction.

## Results

The genome-wide screen with the SBI library was repeated 4 times to obtain a final list of 75 genes as a first selection of possible new host co-factors in CD4 downregulation by Nef. Of these, 22 proteins were confirmed independently with individual Mission consortium vectors in the same cell line. Eight proteins contributed to CD4 downregulation in HIV-1 infected SupT1 cells. The host factors identified show differential effect on CD4 surface levels in SupT1 cells expressing either HIV-1 Vpu or Nef proteins individually, that together determine CD4 levels on infected cells. These proteins are mainly involved in endosomal and trans Golgi network (TGN) trafficking.

## Conclusion

Several host proteins involved in endosomal and TGN trafficking differentially affect Nef or Vpu mediated CD4 down-regulation in HIV1-1 infected cells.

